# Flexible modulation of agonist efficacy at the human A_3 _adenosine receptor by the imidazoquinoline allosteric enhancer LUF6000

**DOI:** 10.1186/1471-2210-8-20

**Published:** 2008-12-12

**Authors:** Zhan-Guo Gao, Kai Ye, Anikó Göblyös, Adriaan P IJzerman, Kenneth A Jacobson

**Affiliations:** 1 Molecular Recognition Section, Laboratory of Bioorganic Chemistry, National Institute of Diabetes and Digestive and Kidney Diseases, National Institutes of Health, Bethesda, Maryland 20892-0810, USA; 2 Division of Medicinal Chemistry, Leiden/Amsterdam Center for Drug Research, Leiden University, PO Box 9502, 2300 RA, Leiden, the Netherlands

## Abstract

**Background:**

A series of 1*H*-imidazo- [4,5-*c*]quinolin-4-amine derivatives, represented by LUF6000 (*N*-(3,4-dichloro-phenyl)-2-cyclohexyl-1*H*-imidazo [4,5-*c*]quinolin-4-amine), are allosteric modulators of the human A_3 _adenosine receptor (AR). Here we studied the modulation by LUF6000 of the maximum effect (E_max_) of structurally diverse agonists at the A_3 _AR stably expressed in CHO cells.

**Results:**

In an assay of [^35^S]GTPγS binding, the E_max _of the A_3 _AR agonist Cl-IB-MECA at the A_3 _AR was lower than that of the non-selective AR agonist NECA. LUF6000 exerted an E_max_-enhancing effect at a concentration of 0.1 μM or higher, and was shown to increase the E_max _of Cl-IB-MECA and other low-efficacy agonists to a larger extent than that of the high-efficacy agonist NECA. Interestingly, LUF6000 converted a nucleoside A_3 _AR antagonist MRS542, but not a non-nucleoside antagonist MRS1220, into an agonist. LUF6000 alone did not show any effect. Mathematical modeling was performed to explain the differential effects of LUF6000 on agonists with various E_max_. A simple explanation for the observation that LUF6000 has a much stronger effect on Cl-IB-MECA than on NECA derived from the mathematical modeling is that NECA has relatively strong intrinsic efficacy, such that the response is already close to the maximum response. Therefore, LUF6000 cannot enhance E_max _much further.

**Conclusion:**

LUF6000 was found to be an allosteric enhancer of E_max _of structurally diverse agonists at the A_3 _AR, being more effective for low-E_max _agonists than for high-E_max _agonists. LUF6000 was demonstrated to convert an antagonist into an agonist, which represents the first example in G protein-coupled receptors. The observations from the present study are consistent with that predicted by mathematical modeling.

## Background

Adenosine receptors (ARs) are G protein-coupled receptors, consisting of A_1_, A_2A_, A_2B _and A_3 _subtypes, activated by the endogenous agonist adenosine and blocked by natural antagonists, such as caffeine and theophylline. A_1 _and A_3 _subtypes are coupled to G_i/o _proteins, while A_2A _and A_2B _subtypes are G_s _protein-coupled. There is growing evidence that they could be promising therapeutic targets in a wide range of conditions [1-3].

Subtype-selective AR agonists have been developed, however, the selectivity for some organs or tissues is nearly unachievable using orthosteric agonists that act directly at the principal ligand binding site of the receptor. This is due to the wide distribution of ARs and, indeed, a number of agonists were discontinued after the initial phases of clinical trials [3-5]. In contrast to directly-acting agonists, allosteric modulators act at a distinct site on the receptor protein to modulate the effect of a native agonist [6-10]. An advantage of an allosteric enhancer of a GPCR over its native, orthosteric activator is that greater selectivity can be achieved. This is due to allosteric sites being generally less conserved than the orthosteric site in a particular receptor family [8]. Furthermore, the allosteric enhancer would enhance the action of the native agonist, but may have no effect of its own on the unoccupied receptor. Thus, the effect of an endogenous agonist, which may be insufficient in a particular disease state, may be magnified in a temporally and spatially specific manner through allosteric modulation.

Allosteric modulation of membrane receptors is best characterized in ligand-gated ion channels. The allosteric enhancer diazepam, which enhances the CNS inhibitory function of the endogenous γ-aminobutyric acid, is a prototypic representative of the benzodiazepines, the most widely prescribed sleep medications. In the GPCR field, cinacalcet, an allosteric enhancer of the calcium-sensing receptor (CaR), has recently been approved for the treatment of secondary hyperparathyroidism in dialysis patients suffering from chronic kidney disease [11]. In the case of ARs, the A_1 _AR has been the most studied in this context, and one of its allosteric enhancers, T62 (2-amino-4,5,6,7-tetrahydrobenzo [b]thiophen-3-yl-(4-chlorophenyl)methanone), has been in clinical trials for the treatment of neuropathic pain. Numerous allosteric enhancers and inhibitors for Class B and Class C GPCRs are also in various phases of clinical trials for treatment of a number of disorders [7,10,12-14].

Allosteric modulators for the A_3 _AR have been recently identified and characterized [15]. One class of these allosteric modulators, including the 1H-imidazo- [4,5-c]quinolin-4-amine derivative DU124183, was found to decrease agonist potency while enhancing its maximum effect (E_max_) [16]. Recently, a new series of the imidazoquinoline derivatives has been synthesized [17]. Several of those allosteric modulators, represented by LUF6000, were found to also enhance E_max _but without affecting agonist potency. Thus, the pharmacological profile as a positive allosteric modulator of the A_3 _AR was superior to that of DU124183. In this study, we extended our previous observations by studying the nature of the potentially flexible modulation by LUF6000 of the agonists with a selection of A_3 _AR agonists having a distribution of E_max _values in A_3 _AR-expressing CHO cells using a [^35^S]GTPγS binding assay [18]. We learned that the degree of allosteric enhancement is dependent on the orthosteric ligand examined, which was quantified using a mathematical model [19], adding further subtlety to this new concept of GPCR regulation.

## Results

### AR agonists and allosteric modulators used in the present study

**Figure 1:**
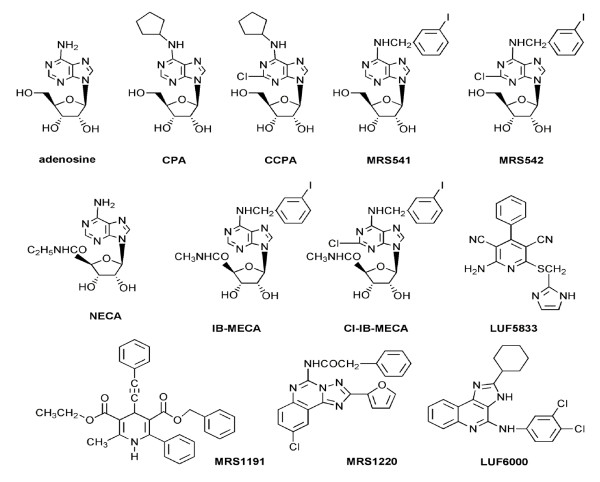
**Chemical structures of the agonists, antagonists, and an allosteric modulator of the A_3 _adenosine receptor used in the present study**.

A number of nucleoside agonists and one non-nucleoside agonist (LUF5833) used in the present study are shown in Figure 1. The 5' -substituted adenosine derivative NECA is a high-efficacy AR agonist. Medium or low efficacy agonists include: an *N*^6^,2,5' -trisubstituted derivative Cl-IB-MECA, a *N*^6^-monosubstituted adenosine derivative MRS541, and a non-nucleoside agonist, LUF5833. In addition to agonists, a nucleoside A_3 _AR antagonist (as defined previously in adenylyl cyclase assays), MRS542, [20] and a non-nucleoside antagonist MRS1220 were also included. The allosteric modulator (Figure 1) used in the present study is the imidazoquinoline derivative LUF6000, which has been shown to retard agonist radioligand dissociation and to increase agonist E_max_, as demonstrated using a cyclic AMP functional assay [17]. LUF6000 was relatively potent as an enhancer of A_3 _AR agonist activity in comparison to its A_3 _AR antagonistic properties. It did not bind appreciably at the other subtypes of ARs.

### Effects of LUF6000 on the Emax at the A_3 _AR of agonists with diverse structures studied with a [^35^S]GTPγS binding assay

**Figure 2:**
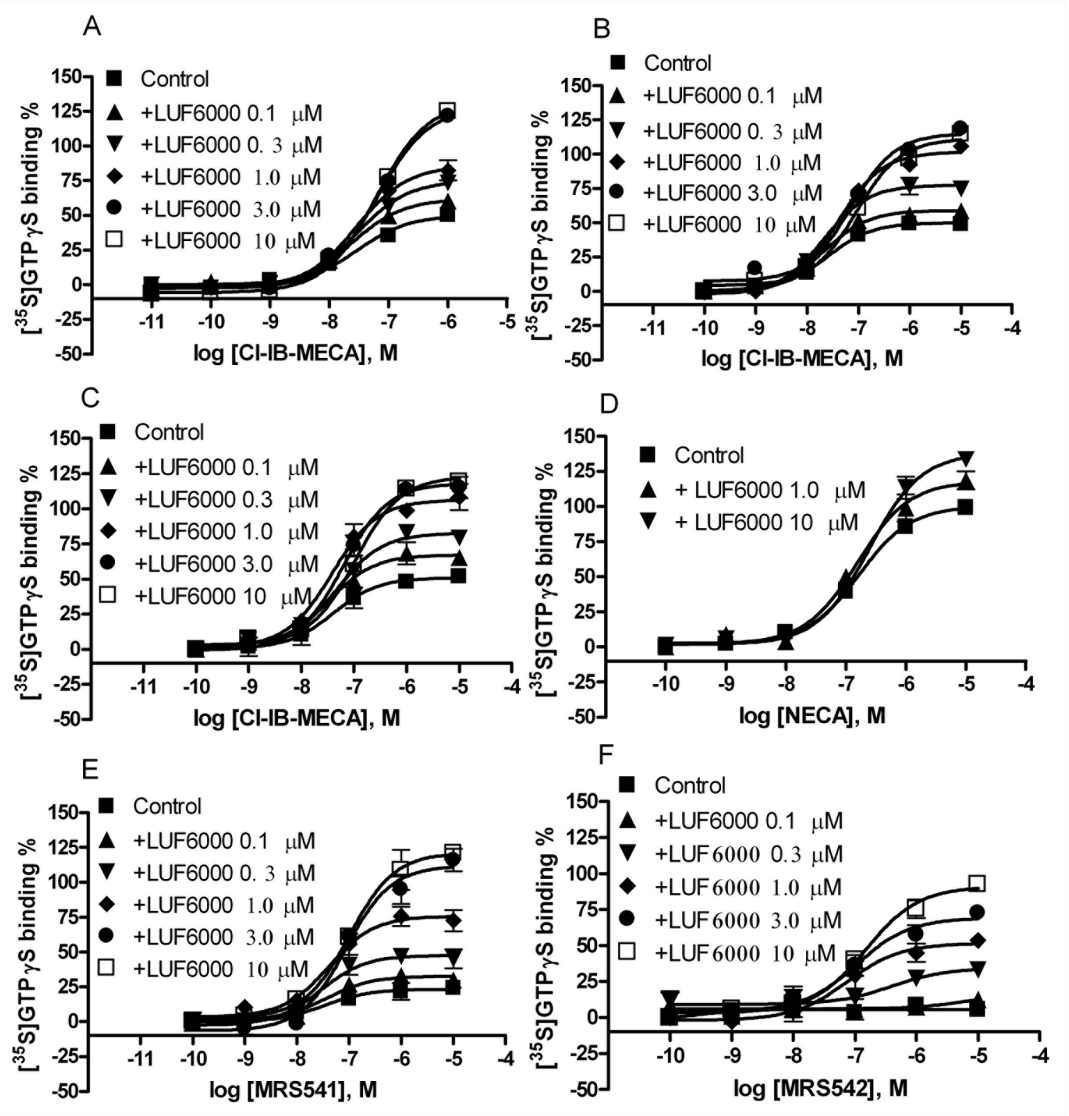
**Effect of LUF6000 on agonist-induced activation of the human A_3 _AR studied with a [^35^S]GTPγS binding assay**. Incubations were started by addition of the membrane suspension (5 μg protein/tube) to the test tubes, and carried out in duplicate for 30 min at 25°C, except for B and C in which LUF6000 or both LUF6000 and an agonist were incubated for 20 min with membranes and other components before the addition of [^35^S]GTPγS (final concentration 0.2 nM). The experimental procedures are described in the Materials and Methods section. Results were from 3–5 independent experiments performed in duplicate. The basal values typically ranged from 800 to 1200 cpm. The maximal values are typically from 2000 to 2500 cpm. A. Cl-IB-MECA. B. Cl-IB-MECA (LUF6000 was incubated with membranes 20 min before the addition of other components). C. Cl-IB-MECA (Both LUF6000 and Cl-IB-MECA were incubated 20 min with membranes before the addition of other ingredients); D. NECA; E. MRS541; MRS542. The maximum stimulation of NECA in the absence of enhancers was expressed as 100%.

The modulation by LUF6000 of the E_max _of diverse agonists at the A_3 _AR was studied using a [^35^S]GTPγS binding assay, which directly reflects activation of G_i/o _proteins. We first compared the E_max_-enhancing effects of LUF6000 under several experimental conditions: (a) addition of the enhancer LUF6000 and an agonist Cl-IB-MECA simultaneously (Figure 2A); (b) pre-incubation of the enhancer LUF6000 with membranes for 20 min before the addition of the agonist to be incubated for another 30 min (Figure 2B); (c) pre-incubation of membranes with both LUF6000 and an agonist for 30 min (Figure 2C). It was found that LUF6000, at the concentration of 0.1 μM or higher, produced a similar enhancement of the E_max _of Cl-IB-MECA under all of these conditions. Thus, the subsequent experiments were performed without the pre-incubation of LUF6000 and/or agonist with membranes. Also, a longer incubation time (90 min) did not cause further stimulation, but increased the non-specific binding (data not shown). The potency and E_max _of Cl-IB-MECA to stimulate [^35^S]GTPγS binding in the absence and presence of various concentrations of LUF6000 are summarized in Table 1.

**Table 1:** Effects of LUF6000 on the potency (-logEC_50_) and maximum effect (E_max_) at the A_3 _AR of structurally diverse ligands measured with a [^35^S]GTPγS binding assay

**Ligands**	**Emax (%)**	**-logEC_50 _(M)**
**Cl-IB-MECA**	50.1 ± 2.4	7.52 ± 0.11
+LUF 0.1 μM	60.4 ± 3.7	7.62 ± 0.12
+LUF 0.3 μM	70.5 ± 5.2	7.54 ± 0.09
+LUF 1.0 μM	82.4 ± 7.3	7.55 ± 0.10
+LUF 3.0 μM	122 ± 3	7.20 ± 0.06
+LUF 10 μM	125 ± 2	7.21 ± 0.06
		
**NECA**	100 ± 3	6.79 ± 0.06
+LUF 1.0 μM	119 ± 7	6.81 ± 0.10
+LUF 10 μM	134 ± 5	6.61 ± 0.07
		
**MRS541**	24.3 ± 2.9	7.48 ± 0.31
+LUF 0.1 μM	30.0 ± 1.7	7.43 ± 0.25
+LUF 0.3 μM	45.0 ± 7.0	7.55 ± 0.22
+LUF 1.0 μM	72.5 ± 7.6	7.39 ± 0.16
+LUF 3.0 μM	116 ± 8	7.06 ± 0.11
+LUF 10 μM	121 ± 6	6.99 ± 0.11
		
**MRS542**	5.6 ± 2.5	NA
+LUF 0.1 μM	16.4 ± 3.1	NA
+LUF 0.3 μM	34.2 ± 5.4	6.41 ± 0.47
+LUF 1.0 μM	51.7 ± 4.1	7.12 ± 0.20
+LUF 3.0 μM	69.2 ± 4.5	7.14 ± 0.15
+LUF 10 μM	91.1 ± 4.1	6.82 ± 0.11
		
**LUF5833**	35.2 ± 5.2	6.02 ± 0.26
+LUF 0.1 μM	63.8 ± 5.2	5.91 ± 0.14
+LUF 1.0 μM	92.7 ± 9.7	5.92 ± 0.18
+LUF 10 μM	102 ± 7.6	5.99 ± 0.13
		
**MRS1220**	NA	NA
+LUF 10 μM	NA	NA

The E_max _of NECA in the absence of LUF6000 was expressed as 100%. Results are expressed as mean ± S.E. LUF, LUF6000; NA, not applicable.

**Figure 3:**
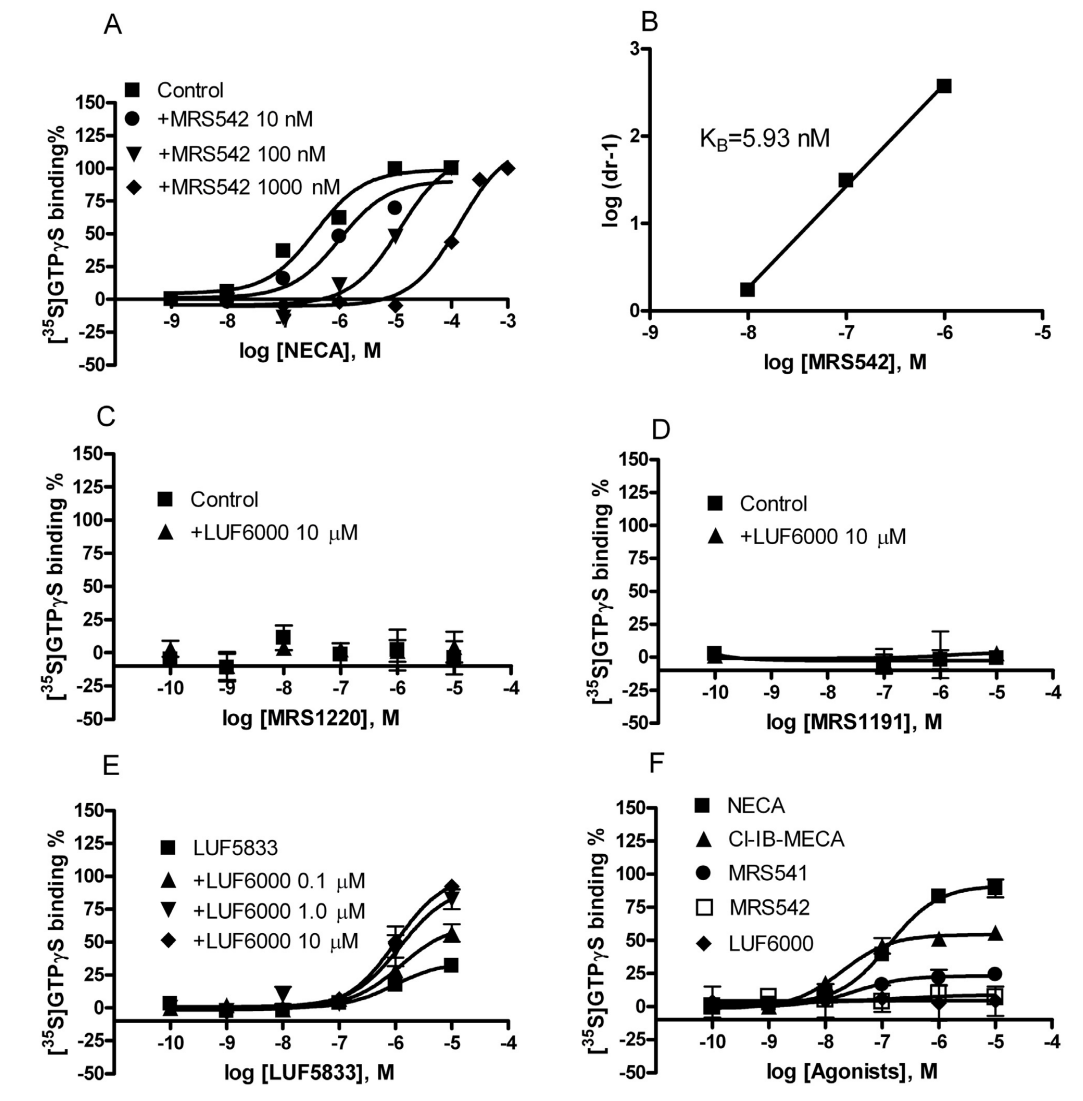
**Effect of LUF6000 on various human A_3 _AR ligands, studied with a [^35^S]GTPγS binding assay**. Incubations were started by addition of the membrane suspension (5 μg protein/tube) to the test tubes, and carried out in duplicate for 30 min at 25°C. The experimental procedures are described in the Materials and Methods section. Results were from 3–5 independent experiments performed in duplicate. The basal values typically ranged from 800 to 1200 cpm. The maximal values are typically from 2000 to 2500 cpm.

LUF6000 had a less pronounced effect on the E_max _of NECA (Figure 2D), but showed a much larger effect on the low-efficacy agonist MRS541 (Figure 2E) compared with its effect on Cl-IB-MECA. MRS542, which has been shown to be an antagonist previously as demonstrated in a cyclic AMP assay [20], was also demonstrated to be an antagonist in the present GTPγS binding assay (Figure 3A and Figure 3B). However, interestingly, MRS542 was converted into an agonist by LUF6000 in a concentration-dependent manner (Figure 2F). Additionally, LUF6000 also enhanced the stimulation of [^35^S]GTPγS binding induced by the non-nucleoside agonist, LUF5833 (Figure 3E), whereas it had no effect on the non-nucleoside antagonists MRS1220 (Figure 3C) and MRS1191 (Figure 3D). LUF6000, at concentrations from 0.1 to 10 μM, did not show any effect on the basal level of A_3 _AR activation in this [^35^S]GTPγS assay (Figure 3F). The potency and E_max _of various ligands in the absence and presence of various concentrations of LUF6000 are summarized in Table 1. The dose-response curves of NECA, Cl-IB-MECA, MRS541, LUF6000 and MRS542 are shown in Figure 3F. Cl-IB-MECA is only partially efficacious compared with NECA in inducing [^35^S]GTPγS binding.

In order to confirm the effect of LUF6000 on the E_max _of the selective agonist Cl-IB-MECA at the A_3 _AR, we further tested its effect on two other A_3 _AR selective agonists, IB-MECA and inosine. It was demonstrated that IB-MECA was almost as efficacious as Cl-IB-MECA, and LUF6000 showed similar effect on these two A_3 _AR agonists. Inosine only showed a low E_max _(< 10% of that of NECA). However, in the presence of 10 μM LUF6000, the E_max _of inosine was shown to be approximately 80% of the E_max _of NECA.

**Figure 4:**
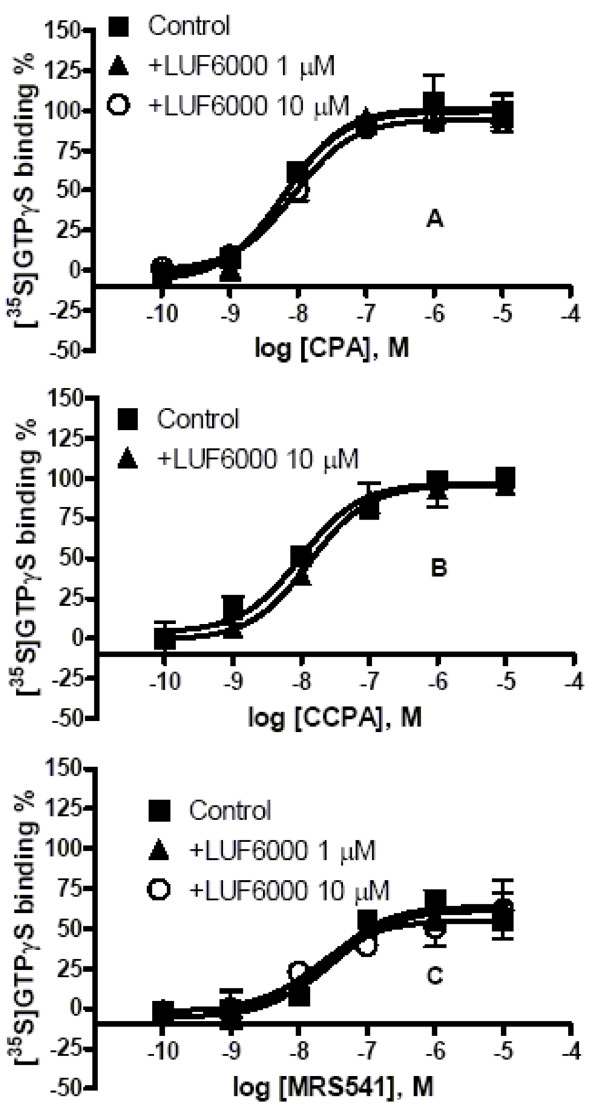
**Effect of LUF6000 on agonist-induced activation of the human A_1 _AR studied with a [^35^S]GTPγS binding assay**. Incubations were started by addition of the membrane suspension (5 μg protein/tube) to the test tubes, and carried out in duplicate for 30 min at 25°C. Results were from 3 independent experiments performed in duplicate. The basal values ranged from 700 to 1000 cpm. The maximal values are typically from 1500 to 1800 cpm.

Unlike its effect on the A_3 _AR, LUF6000 did not affect the E_max _of two high-efficacy agonists CPA and CCPA or a low efficacy agonist MRS541 to activate the A_1 _AR (Figure 4).

### Mathematical modeling

The equations from Hall [19] were used to simulate various experimental curves and to derive conditions in which E_max _and EC_50 _vary. Parameters in these equations are as follows. L is the receptor isomerization constant (the ratio of receptor in the active state over the inactive state). K is the equilibrium association constant of ligand A. M is the equilibrium association constant of ligand B (allosteric modulator). α is the intrinsic efficacy of ligand A. β is the intrinsic efficacy of ligand B. γ is the binding cooperativity between A and B. δ is the activation cooperativity between A and B. L is only related to the receptor. The allosteric modulator brings three parameters, β, γ and δ, to the system. The proportion of receptors in the active state is:

$$\frac{\left[R_{active}\right]}{\left[R_{T}\right]}=\frac{\left[R^{*}]+[AR^{*}]+[R^{*}B]+[AR^{*}B\right]}{\left[R]+[R^{*}]+[AR]+[AR^{*}]+[RB]+[R^{*}B]+[ARB]+[AR^{*}B\right]}$$

This can be restated as:

$$\frac{\left[R_{active}\right]}{\left[R_{T}\right]}=\frac{L(1+\alpha K[A]+\beta M[B](1+\alpha \gamma \delta K[A])}{1+L+M[B][1+\beta L)+K[A](1+\alpha L+\gamma M[B](1+\alpha \beta \delta L)}$$

Since LUF6000 did not affect the function of the A_3 _AR when given alone, we concluded it has neutral intrinsic efficacy (β = 1). Next we derived the conditions under which the maximal effect (E_max_) and observed potency of an agonist can change in the presence of an allosteric modulator. When there is a saturating concentration of agonist in the absence of allosteric modulator, the proportion of active receptor populations over all receptors is given by:

$$\frac{{\left[R\right]}_{active}^{[A]\to \infty ,[B]=0}}{{\left[R\right]}_{T}}=\frac{\alpha L}{1+\alpha L}$$

When there is also an excess amount of allosteric modulator, the proportion is given as:

$$\frac{{\left[R\right]}_{active}^{[A]\to \infty ,[B]\to \infty ,\beta =1}}{{\left[R\right]}_{T}}=\frac{\alpha L\delta }{1+\alpha L\delta }$$

Comparing these two equations, we learned that if

*δ *> 1, the maximum response increases;

*δ *= 1, the maximum response remains unchanged;

*δ *< 1, the maximum response decreases;

Based on Hall's model of allosteric modulation, the agonist affinity ratio in the presence and absence of an allosteric modulator with neutral intrinsic efficacy is:

$$\frac{{\left[A\right]}_{active}^{[B]\to \infty ,\beta =1}}{{\left[A\right]}_{50}^{[B]=0}}=\frac{1+\alpha L}{\gamma (1+\alpha L\delta )}$$

Thus, if

$$\gamma >\frac{1+\alpha L}{1+\alpha L\delta },\text{ the potency increases }(\text{curve shifts to the left});$$

$$\gamma =\frac{1+\alpha L}{1+\alpha L\delta },\text{ the potency remains unchanged;}$$

$$\gamma <\frac{1+\alpha L}{1+\alpha L\delta },\text{ the potency decreases (curve shifts to the right);}$$

We also analyzed the condition in which an allosteric modulator converts a neutral antagonist or even an inverse agonist into an agonist. In the presence of excess amount of an allosteric modulator, the window, i.e. difference in E_max_, observed in the absence and presence of a ligand in the orthosteric site is

$$\frac{{\left[R\right]}_{active}^{[A]=\infty ,[B]\to \infty ,\beta =1}}{{\left[R\right]}_{T}}-\frac{{\left[R\right]}_{active}^{[A]=0,[B]\to \infty ,\beta =1}}{{\left[R\right]}_{T}}=\frac{L\alpha \delta }{1+L\alpha \delta }-\frac{L}{1+L}$$

When *αδ *> 1, agonism is observed. This implies that when δ, the parameter for activation cooperativity, is large enough, an allosteric modulator can convert a neutral antagonist and an inverse agonist into agonists.

**Figure 5:**
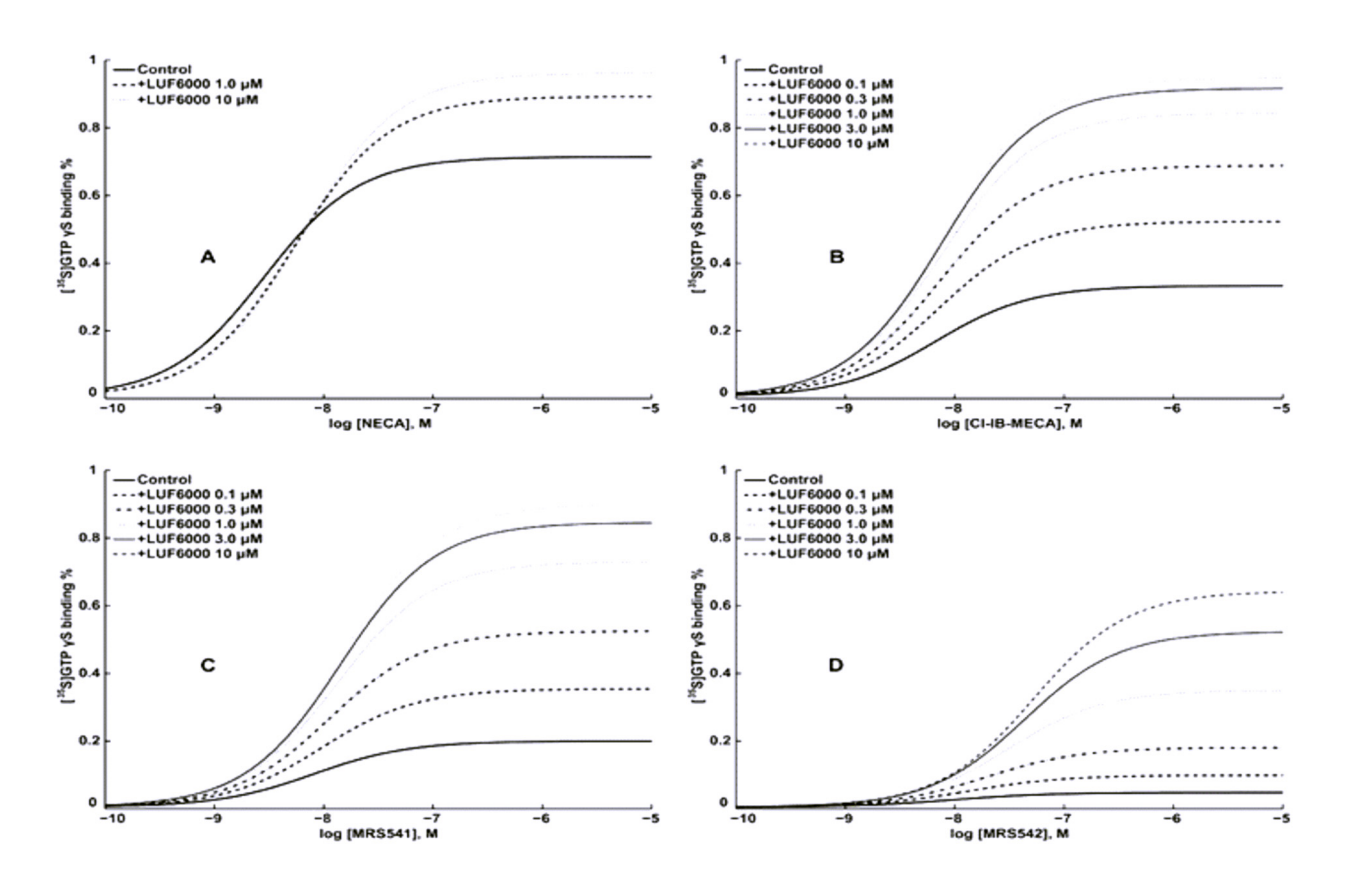
**Simulation of concentration-effect curves with MatLab**. The equations from Hall (2000) were used to derive conditions that vary in efficacy and potency. The parameters in these equations are explained and discussed in the text. Parts A, B, C and D mimic the experimental concentration-effect curves of Figures 2D, 2C, 2E and 2F, respectively.

We simulated a number of experimental concentration-effect curves as depicted in Figure 2, reflecting the use of different agonists (Figure 5). We only varied the intrinsic efficacy (α) of the orthosteric ligand A, i.e. for NECA, Cl-IB-MECA, MRS541 and MRS542 α values were set to 500, 100, 50 and 5, respectivly. The values of the other parameters were as follows: *L *= 0.005; *K *= 1 × 10^8^; *M *= 5 × 10^6^; *β *= 1; *γ *= 0.05; *δ *= 50; As is evident from Figure 5, these settings allow for a simulation of concentration-effect curves that closely resemble the ones obtained experimentally.

## Discussion

The result that LUF6000 alone did not show any agonist activity indicates that, under physiological conditions where the concentration of native adenosine is relatively low, LUF6000 should have no effect. The fact that LUF6000 showed significant enhancement of the E_max _of agonists, suggests that it could enhance agonist efficacy under some pathophysiological conditions such as ischemia and inflammation where concentrations of endogenous agonists are highly elevated.

It is interesting that LUF6000 was shown to enhance the E_max _of low-efficacy agonists to a larger extent than that of the high-efficacy agonists. Furthermore, LUF6000 was demonstrated to be able to convert a nucleoside antagonist MRS542 into an agonist. The effect did not occur with the non-nucleoside antagonists MRS1220 and MRS1191. This may suggest that the nucleoside antagonist may have a small residual activity that would normally be below the detection threshold but could be greatly magnified in the presence of LUF6000.

The ability of GPCRs to adopt various conformational states, each with distinct pharmacological properties, and the phenomenon of stabilization of a specific conformation of a GPCR by an allosteric enhancer have been well documented [9,21].

Activation of a given GPCR could be accomplished with an allosteric agonist, in addition an orthosteric agonist [10,22-28]. Conversion of antagonists into agonists has been demonstrated previously by site-directed mutagenesis of GPCRs [29-33] or by chemical modification of the antagonists [20,34]. However, to our knowledge, there has not been a report regarding this type of conversion by an allosteric modulator, although an example of the conversion of an agonist into antagonist by an allosteric modulator has been reported previously [35]. Thus, it is likely that our finding that an allosteric enhancer can convert an apparent antagonist into an agonist may be generalized to other GPCRs. A better understanding of the conformational states and the mechanisms of GPCR activation will facilitate the design of more effective and selective drugs.

A simple R⇔R* model representing the two conformational states (inactive R, and active R*) of the receptor has been previously used to rationalize the effect of the A_1 _AR enhancer PD81,723 [36]. PD81,723 alone behaves as an allosteric agonist, promoting the A_1 _AR to its active conformation (R*) and, thus, potentiates the A_1 _AR constitutive receptor activity. However, LUF6000 alone does not activate the A_3 _AR. Thus, unlike PD81,723, which shifts the receptor to the R* state directly, LUF6000 presumably does not cause such a shift.

An extension of the two-state model of receptor activation has been described by Hall [19] to account for the allosteric modulators affecting the agonist potency as well as the intrinsic efficacy of agonists. Hall [19] suggested that the most suitable assay system may be one with very low receptor expression in which even highly efficacious agonists are unable to fully activate the signal transduction cascade. We have previously described that our cyclic AMP functional assay system is an ideal model for the characterization of the functional aspects of this class of allosteric modulators, especially the characterization of their effects on maximal agonist efficacy due to the less-than-complete inhibition of adenylyl cyclase by A_3 _AR agonists [16,17]. The present study extended the previous one by using the newly synthesized enhancer LUF6000 and structurally diverse agonists and by testing the stimulation of GTPγS binding. It was found that in the current [^35^S]GTPγS binding assay system, none of the agonists (previously assumed to be full or partial agonists mainly based on a cyclic AMP functional assay) were fully efficacious, as the allosteric enhancer LUF6000 was able to enhance the E_max _of all agonists used. Thus, the current [^35^S]GTPγS binding assay may represent the most suitable assay system as Hall suggested [19], since even highly efficacious agonists are unable to fully activate the signal transduction cascade. The observations from the present study are consistent with that predicted by Hall's model, and thus, the equations from Hall [19] were used to simulate various experimental curves from the present study and to derive conditions in which E_max _and potency vary.

For the mathematical modeling, two independent parameters are necessary to simulate various effects on efficacy and potency. The binding cooperativity shifts the potency of the agonist. The activation coöperativity changes the E_max _that the agonist can achieve, as well as the potency of the agonist. By contrast, the simple model of Ehlert for allosteric modulation [37] uses only one parameter to describe the property of allosteric modulation, so that it cannot simulate the current experimental results. The simple model of allosteric modulation can only generate curves with increased or decreased potency and efficacy at the same time.

As demonstrated in the present study, NECA is more efficacious than Cl-IB-MECA in stimulating G proteins as measured in the [^35^S]GTPγS binding assay. An explanation for the observation that LUF6000 has a much stronger effect on Cl-IB-MECA than on NECA derives from the mathematical modeling. NECA has a relatively strong intrinsic efficacy, such that the response is already close to the maximum response. Therefore, LUF6000 cannot enhance E_max _much further.

## Conclusion

LUF6000 showed a flexible modulatory effect of efficacy depending on the E_max _of a given nucleoside derivative, being more effective for low-E_max _agonists than for high- E_max _agonists. The fact that LUF6000 did not show any effect on the basal receptor activity, yet showed an enhancement of agonist activity, suggests that such enhancers could be safer drugs than orthosteric agonists. The observations from the present study are consistent with the quantitative prediction using Hall's model. A simple explanation for the observation that LUF6000 has a much stronger effect on Cl-IB-MECA than on NECA derived from the mathematical modeling is that NECA has relatively strong intrinsic efficacy, such that the response is already close to the maximum response. Therefore, LUF6000 cannot enhance E_max _much further. The finding that an antagonist of the A_3 _AR can be converted into an agonist may represent a novel mechanism of GPCR activation and may be generalized to other GPCRs.

## Methods

### Materials

Adenosine deaminase was obtained from Worthington Biochemical Corp. (Lakewood, NJ). NECA (adenosine-5' -*N*-ethyluronamide), CCPA (2-chloro-*N*^6^-cyclopentyladenosine) and Cl-IB-MECA (2-chloro-*N*^6^-(3-iodobenzyl)adenosine-5' -*N*-methyluronamide) were from Sigma (MO, USA). LUF6000 (*N*-(3,4-dichloro-phenyl)-2-cyclohexyl-1*H*-imidazo [4,5-*c*]quinolin-4-amine) and LUF5833 (2-aminophenyl-6-(1*H*-imidazol-2-ylmethylsulfanyl)-pyridine-3,5-dicarbonitrile) were synthesized at Leiden/Amsterdam Center for Drug Research (Leiden, The Netherlands). MRS541 (*N*^6^-(3-iodobenzyl)adenosine) and MRS542 (2-chloro-*N*^6^-(3-iodobenzyl)adenosine) were synthesized at NIDDK, National Institutes of Health (Bethesda, MD, USA). All compounds were stored at -20°C as DMSO solutions. DMSO was added to the controls in all experiments. [^35^S]GTPγS (1068 Ci/mmol) was from Amersham (Buckinghamshire, UK). All other compounds, reagents, or solutions were obtained from standard commercial sources and were of analytical grade.

### Cell Culture

Chinese hamster ovary (CHO) cells stably expressing A_1 _or A_3 _AR were maintained at 37°C with 5% CO_2 _in a 1:1 mixture of Dulbecco's modified Eagle's medium (DMEM) and Ham's F12 medium, supplemented with 10% fetal bovine serum (FBS), 100 units/ml penicillin, 100 μg/mL streptomycin, and 2 mM glutamine.

### [^35^S]GTPγS binding assay

The preparation of membranes from CHO cells stably expressing human A_1 _or A_3 _AR was as previously described [18]. [^35^S]GTPγS binding was measured in 200 μl buffer containing 50 mM Tris HCl (pH 7.4), 1 mM EDTA, 1 mM MgCl_2_, 1 μM GDP, 1 mM dithiothreitol, 100 mM NaCl, 3 Units/ml adenosine deaminase, 0.2 nM [^35^S]GTPγS, 0.004% 3- [(3-cholamidopropyl) dimethylammonio]propanesulfonate (CHAPS), and 0.5% bovine serum albumin. Incubations were started by addition of the membrane suspension (5 μg protein/tube) to the test tubes, and carried out in duplicate for 30 min at 25°C. The reaction was stopped by rapid filtration through Whatman GF/B filters, pre-soaked in 50 mM Tris HCl, 5 mM MgCl_2 _(pH 7.4) containing 0.02% CHAPS. The filters were washed twice with 3 ml of the buffer mentioned before, and retained radioactivity was measured using liquid scintillation counting. Non-specific binding of [^35^S]GTPγS was measured in the presence of 10 μM unlabelled GTPγS.

### Simulation of concentration-effect curves using MatLab

A mathematical model for allosteric modulation [19] was implemented in MatLab (version 7.1), a software package for technical computing. A graphic user interface was composed to facilitate parameter input and to yield output in the form of simulated curves.

#### Data analysis

Functional parameters were calculated using Prism 4.0 software (GraphPAD, San Diego, CA, USA). Data were expressed as mean ± sem. Student's t-test or ANOVA was used where appropriate for statistical analysis.

## Abbreviations

LUF6000: *N*-(3,4-dichloro-phenyl)-2-cyclohexyl-1*H*-imidazo [4,5-*c*]quinolin-4-amine; Cl-IB-MECA: 2-chloro-*N*^6^-(3-iodobenzyl)-adenosine-5' -*N*-methyluronamide; MRS541: *N*^6^-(3-iodobenzyl)adenosine; MRS542: 2-chloro-*N*^6^-(3-iodobenzyl)adenosine; LUF5833: 2-aminophenyl-6-(1*H*-imidazol-2-ylmethylsulfanyl)-pyridine-3,5-dicarbonitrile; CCPA: 2-chloro-*N*^6^-cyclopentyladenosine; MRS1191: 1,4-dihydro-2-methyl-6-phenyl-4-(phenylethynyl)-3,5-pyridinedicarboxylic acid, 3-ethyl 5-(phenylmethyl) ester; MRS1220: *N*- [9-chloro-2-(2-furanyl)[1,2,4]triazolo [1,5-c]quinazolin-5-yl]benzeneacetamide.

## Authors' contributions

ZGG designed and carried out the pharmacological experiments and prepared the manuscript. KY performed the mathematical modeling. AG synthesized the allosteric enhancer LUF6000. IJAP and KAJ participated in design and coordination and helped to prepare the manuscript, and have given the approval of the final version to be published. All authors read and approved the final manuscript.
